# Normative data and correlation parameters for vessel density measured by 6 × 6-mm optical coherence tomography angiography in a large chinese urban healthy elderly population: date from the Beichen eye study

**DOI:** 10.1186/s12886-024-03561-z

**Published:** 2024-07-19

**Authors:** Shuzhan Xu, Fei Gao, Rong Luan, Yuqing Liu, the Beichen Eye Study Group, Xiaorong Li, Juping Liu

**Affiliations:** Tianjin Key Laboratory of Retinal Functions and Diseases, Tianjin Branch of National Clinical Research Center for Ocular Disease, Eye Institute and School of Optometry, Tianjin Medical University Eye Hospital, Nankai District, 251 Fukang Rd, Tianjin, 300384 China

**Keywords:** Optical coherence tomography angiography, Retinal vessel density, Choriocapillaris, Observational study, Epidemiology, Normative data

## Abstract

**Background:**

To establish a normative database for macular vessel density (VD) measured by optical coherence tomography angiography (OCTA) and explore the parameters related to the VD.

**Methods:**

An observational study in epidemiology. 5840 healthy elderly participants in Beichen district, Tianjin, China underwent detailed ophthalmic and systemic examinations. OCTA was performed in all subjects using a 6 × 6-mm line scan mode centered on the macula and the built-in software was used to quantify VD and stratify the retina.

**Results:**

One thousand four hundred sixty-one healthy elderly citizens (30.4% men) were included, with a median age of 60.0 years (8.0 years) and an age range of 50 to 87 years.VDs in the different plexuses: superficial capillary plexus (SCP) 43.9% (3.2%), deep capillary plexus (DCP) 44.3% (2.8%), outer capillary plexus (OCP) 21.9% (5.9%), choriocapillaris (CC) 52.1% (1.4%). 90% medical reference range of the VDs at different plexuses was reported. Age was correlated with the VDs of each capillary plexus. Sex was correlated with the VDs of DCP and OCP, and the VDs of DCP (*p* < 0.001) and OCP (*p* = 0.015) in women were higher than that in men. After age and sex adjustment, choroid average thickness was positively correlated with VDs of SCP (*R* = 0.067, *p* = 0.010) and DCP (*R* = 0.108, *p* < 0.001), ganglion cell layer (GCL) average thickness (*R* = 0.072, *p* = 0.006) was positively correlated with the VD of OCP, best-corrected visual acuity (BCVA) (*R* = 0.082, *p* = 0.002) was positively correlated with the VD of CC.

**Conclusions:**

In this study, the normative VD database of the Chinese urban healthy elderly population measured by the OCTA was established, and parameters related to the VD of each capillary plexus were analyzed, providing new ideas for the future study of the relationship between macular VD and disease.

**Trial registration:**

The Beichen Eye Study had been registered on the Chinese Clinical Trial Registry website (registry number: ChiCTR2000032280) on April 25, 2020.

## Background

The macula is one of the most metabolic tissues in the human body [1]. The macula capillary network plays an important role in maintaining visual acuity and normal macular structure [2]. Spaide et al. speculated that some diseases, especially those changing macular perfusion, may have different effects on one layer of the vascular network from the other [3]. Therefore, quantifying macular vessel density (VD) and structural in vivo is helpful to understand its physiological state and monitor the progress of the disease, and also has important reference significance for the study of retinal disease. Optical coherence tomography angiography (OCTA) is a new technique to reconstruct the retinal capillary network by detecting changes in blood flow signals [4]. It can visualize the capillary network of each layer of the retina without the need to inject the dye [5, 6]. Compared with traditional retinal vessel imaging techniques such as fundus fluorescein angiography (FFA), OCTA is safer, simpler, and faster, and can quantify the density and perfusion of retinal capillaries. In recent years, it has been widely used for screening retinal diseases [7].

Previous studies have shown that OCTA has good repeatability and clinical applicability in the diagnosis of retinal diseases and choroidal diseases [8, 9]. The present research on OCTA mostly focuses on the relationship between OCTA parameters and retinal or choroidal diseases such as diabetic retinopathy (DR) [10] and age-related macular degeneration (AMD) [11]. However, studies on normative OCTA parameters in large-scale healthy populations are still rare. The normal values of many parameters of OCTA and their relationship with other ocular and systemic parameters are mostly unknown so far. However, if a parameter is used to diagnose a disease, the normal change of the parameter and its associated parameters must be known. At present, there are many limitations in those studies of the normative data of OCTA parameters in healthy people [12–16]. For example, the sample sizes of those studies were usually small, the subjects of those studies were not from the population, and most of the studies used 3 × 3-mm OCTA equipment, resulting in a narrow view field in those studies. In addition, the contents and values of the data reported in many studies are different due to different manufacturers of OCTA devices and different retinal stratification criteria [4].

In the present study, we established a normative database of VD measured by 6 × 6-mm OCTA in the superficial capillary plexus (SCP), deep capillary plexus (DCP), outer capillary plexus (OCP), and choriocapillaris (CC) plexuses of the macula in a large Chinese urban healthy elderly population, and its correlation with other ocular parameters and systemic parameters were also analyzed.

## Method

### Study population

The study for eye diseases among Beichen Eye Study (BCES) was conducted in the Beichen District, Tianjin, China from June 2020 to February 2022. Beichen district is located in the north of the central district of Tianjin, China, which is an inner-suburb between the central districts and outer-suburb as well as rural areas. As a transition region, Beichen district experienced rapid urbanization during the past 30 year [17]. Five thousand eight hundred forty participants greater than 50 years were recruited by a multi-stage cluster random sampling method and underwent a single study visit nested within BCES, at which visual function, systemic parameters, and retinal imaging were captured. The study adhered to the Declaration of Helsinki and was approved by the institutional review board of Tianjin Medical University Eye Hospital (2019ky-22). Written informed consent was signed by all participants. The BCES had been registered on the Chinese Clinical Trial Registry website (registry number: ChiCTR2000032280).

**Fig. 1:**
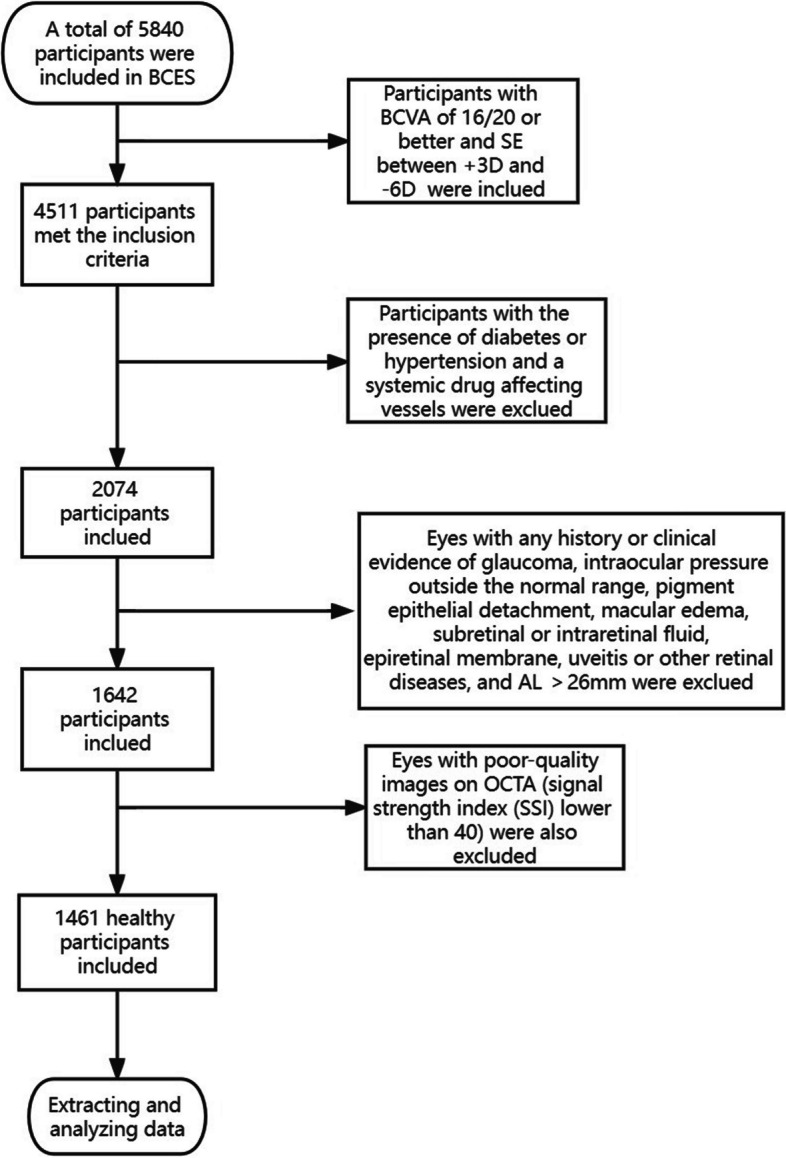
Screening process of healthy subjects

Participants who had a best-corrected visual acuity (BCVA) of 0.10 (logMAR) or better and spherical equivalent (SE) between + 3D and -6D were included in this cross-sectional analysis. Exclusion criteria were: any history or clinical evidence of glaucoma, pigment epithelial detachment, macular edema, subretinal or intraretinal fluid, epiretinal membrane, uveitis or other retinal diseases, presence of diabetes or hypertension, previous ocular surgery or laser photocoagulation, intraocular pressure (IOP) outside the normal range, axial length (AL) > 26 mm, a systemic drug that affects vessels. Eyes with poor-quality images on OCTA (signal strength index lower than 40) were also excluded (Fig. 1).

### Examination

All examinations were performed at the community hospital or residential committee to which the participants belonged. The compound tropicamide eye drops were used for pupil dilation of all participants before the examination. The protocol included a detailed assessment of general health status, questionnaires, and the provision of blood samples for laboratory testing, all of which were performed by physicians, nurses, and optometrists. The examination included anthropometry, ocular biometry, visual acuity, swept-source anterior segment optical coherence tomography (OCT), slit lamp examination, tonometry, fundus photography, nonmydriatic 200° ultra-wide-field scanning laser ophthalmoscopy (SLO), OCT, OCTA, gonioscopy, and visual field test.

### OCTA and quantitative detection of VD

**Fig. 2:**
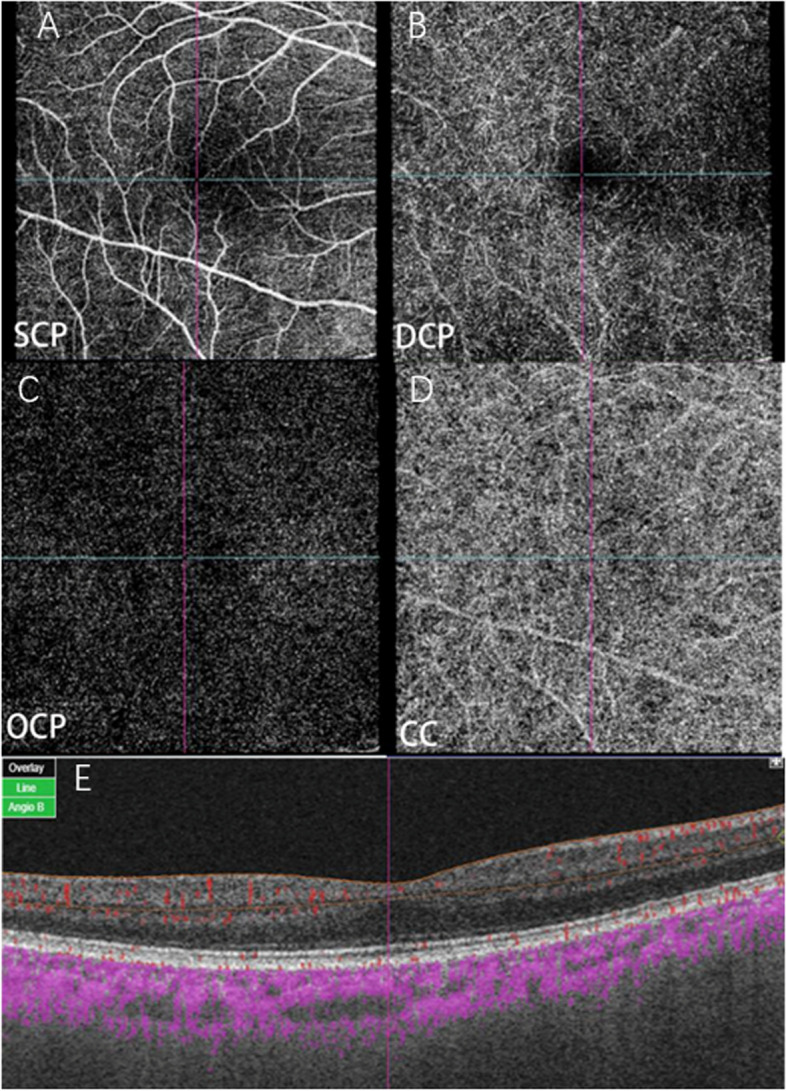
OCTA 6 × 6 mm scan showing macular vessel density in a healthy 60-year-old female with visual acuity of 0 (logMAR). **A** macular vessel density map of superficial capillary plexus (SCP). **B** macular vessel density map of deep capillary plexus (DCP). **C** macular vessel density map of outer capillary plexus (OCP). **D** macular vessel density map of choriocapillaris (CC). **E** coregistered OCT B-scan

OCTA (Topcon Corporation, Tokyo, Japan) images of the bilateral macula were obtained after dilation. The device used a central wavelength of 1050 nm and acquired 100,000 A-scans per second scan. OCTA scans were performed in all subjects using a 6 × 6-mm line scan mode centered on the macula. By using the default automatic stratification function of Triton, the retina is divided into four layers: SCP, DCP, OCP, and CC (Fig. 2). SCP was defined as 2.6 μm below the internal limiting membrane (ILM) to 15.6 μm below the inner plexiform layer (IPL) / inner nuclear layer (INL). DCP was defined from 15.6 μm below the IPL / INL to 70.2 μm below the IPL. OCP was defined as 70 μm from IPL to 30 μm from the retinal pigment epithelium (RPE). The CC was defined as from 0 μm to 10.4 μm below the basement membrane (BM). Manually check the image quality. Manually check the image quality and adjust the center detection area to the macula if the macula is not in the center of the OCTA scan.Vessel density was defined as the percentage of the sample area occupied by the lumen of the vessel after image binarization reconstruction by the built-in software. According to the early treatment diabetic retinopathy study (ETDRS), the macular area was divided into five regions: central, superior, inferior, nasal, and temporal. The VDs were obtained and recorded by IMAGENET 6.0 automatic detection tool, and the VDs were entered into the database.

### Swept-source OCT (SS-OCT) imaging

The following scan patterns of SS-OCT (Topcon Corporation, Tokyo, Japan) were performed: linear B-scan (12 mm in length) centered on the fovea at 0°; 3D macula map covering a central area of a 7 mm × 7 mm scan mode to image the retina during a 1.3-s scan time, with a scan density of 512 A-scans × 512 B-scans. The built-in software of SS-OCT could automatically identify the boundary of each layer of the retina and report the retinal nerve fiber layer (RNFL) thickness, retina average thickness, macular ganglion cell layer (GCL) average thickness and choroid average thickness. The results of the automatic segmentation were evaluated by experts, manually adjusted if segmentation errors occur.

### Statistical analysis

Data were analyzed using SPSS software version 26 (SPSS Inc, Chicago Illinois). Only participants ' right eye data were included in the study. All quantitative variables were reported as median (interquartile range) after confirming the non-normality with the Kolmogorov–Smirnov test. The percentile method was used to determine the 90% medical reference range of macular VDs at different sexes, retinal plexuses and age groups. Mann–Whitney test was used to compare parameters between different sexes. Kruskal–Wallis H test was used to compare the VDs of different parafoveal subfields in the same retinal capillary plexus and the VDs of different age groups. Post hoc test was used for pairwise comparison and the Bonferroni was used for adjusting the *p*-value. The Spearman correlation test was used in the correlation analysis. Correlations between macular vascular density and age, sex, diastolic pressure, systolic pressure, Body mass index (BMI), AL, SE, BCVA, IOP, RNFL thickness, retina average thickness, GCL average thickness and choroid average thickness were analyzed. Partial correlations with the sex and age adjustments were used to study the relationship among those parameters. A *P* value less than 0.05 was considered statistically significant.

## Result

A total of 5840 individuals ranged from 50 to 87 years old were initially included in the BCES. Lastly, after the inclusion and exclusion criteria screening mentioned previously, 1461 eyes of 1461 participants were included in this study (444 eyes of men, 30.4%) and the median age was 60.0 years (8.0 years). The median VD of SCP was 43.9% (3.2%), the median VD of DCP was 44.3% (2.8%), the median VD of OCP was 21.9% (5.9%), and the median VD of CC was 52.1% (1.4%). Comparing the main characteristics between different sexes, it was found that there were significant differences in age, diastolic blood pressure, systolic blood pressure, BMI, AL, mean retinal thickness, and the VD of DCP and OCP. The main characteristics of the study participants were presented in Table 1.

**Table 1:** Demographics, OCT- and OCTA-based measures, and ocular characteristics of the study sample

Parameters	Total	Men	Women	P
N (%)	1461	444(30.4%)	1017(69.6%)	
Age (years)	60.0 (8.0)	62.0 (9.0)	58.0 (8.0)	< 0.001***
Ethnic (Han) (N, %)	1438(98.4%)	438(98.6%)	1000(98.3%)	0.429
Personal income(≥ 5000 yuan/month) (N, %)	564(38.6%)	202(45.5%)	362(35.6%)	< 0.001***
Educational level (higher than primary school) (N, %)	1169(80.0%)	343(77.3%)	826(81.2%)	0.075
Diastolic pressure (mmHg)	133.0 (24.5)	136.0 (24.0)	131.0 (25.0)	< 0.001***
Systolic pressure (mmHg)	84.0 (13.0)	87.0 (14.8)	82.0 (12.0)	< 0.001***
BMI (kg/m^2^)	25.2 (4.2)	26.0 (4.3)	24.9 (4.1)	< 0.001***
BCVA (logMAR)	0 (0)	0 (0.1)	0 (0)	0.176
SE (D)	0 (0.8)	0 (0.8)	0 (0.8)	0.548
AL (mm)	23.1 (1.1)	23.4 (0.8)	22.9 (1.0)	< 0.001***
IOP (mmHg)	16.0 (4.0)	15.7 (2.7)	16.0 (4.0)	0.663
RNFL thickness (μm)	33.8 (4.7)	33.7 (5.2)	33.9 (4.6)	0.566
Retina average thickness (μm)	274.2 (17.3)	275.3 (17.6)	273.8 (17.4)	0.016*
GCL average thickness (μm)	71.5 (6.7)	71.4 (5.2)	71.6 (6.4)	0.568
Choroid average thickness (μm)	205.7 (53.0)	207.4 (56.3)	204.9 (51.4)	0.600
SCP (%)	43.9 (3.2)	43.7 (3.0)	43.9 (3.4)	0.336
DCP (%)	44.3 (2.8)	43.9 (2.9)	44.5 (2.9)	< 0.001***
OCP (%)	21.9 (5.9)	21.3 (6.1)	22.1 (5.7)	0.015*
CC (%)	52.1 (1.4)	52.1 (1.3)	52.1 (1.4)	0.468

Data are median (interquartile range). *p ≤ 0.05 **p ≤ 0.01 ***p ≤ 0.001

*Abbreviations*: *BMI* Body mass index, *BCVA* Best-corrected visual acuity, *SE* Spherical equivalent, *AL* Axial length, *IOP* Intraocular pressure, *RNFL* Retinal nerve fiber layer, *GCL* Ganglion cell layer, *SCP* Superficial capillary plexus, *DCP* Deep capillary plexus, *OCP* Outer capillary plexus, *CC* choriocapillaris

90% medical reference ranges of the VDs were 39.4–47.6% at the level of SCP, 40.1–47.7% at the level of DCP, 15.6–29.1% at the level of OCP, and 49.9–54.3% at the level of CC (Table 2). Participants were grouped by age, 50–59 years old as group 1, 60–79 years old as group 2, and 80–90 years old as group 3. The 90% medical reference ranges of different age groups and different sexes were reported respectively (Table 2). The VDs of SCP(p < 0.001), DCP(p < 0.001) and OCP(*p* = 0.001) were statistically significant different in different age groups. The post hoc test showed that except for age group 1 and group 2 of OCP (*p* = 1.000), the VDs of SCP, DCP and OCP in different age groups were statistically significant different.

**Table 2:** 90% medical reference range of the VDs in different sex groups and age groups of the study sample

	SCP (%)	DCP (%)	OCP (%)	CC (%)
Total	39.4-47.6	40.1-47.7	15.6-29.1	49.9-54.3
Man	39.5-47.4	40.1-46.8	14.9-29.4	50.0-54.0
Woman	39.4-47.7	40.1-47.9	15.9-29.1	49.9-54.4
50-59 (years)	39.7-48.0	40.8-47.7	15.5-28.8	50.0-54.3
60-79 (years)	39.4-47.3	39.8-47.7	15.6-29.3	50.0-54.3
80-90 (years)	37.8-46.4	37.6-46.6	16.7-30.5	49.3-54.1

**Fig. 3:**
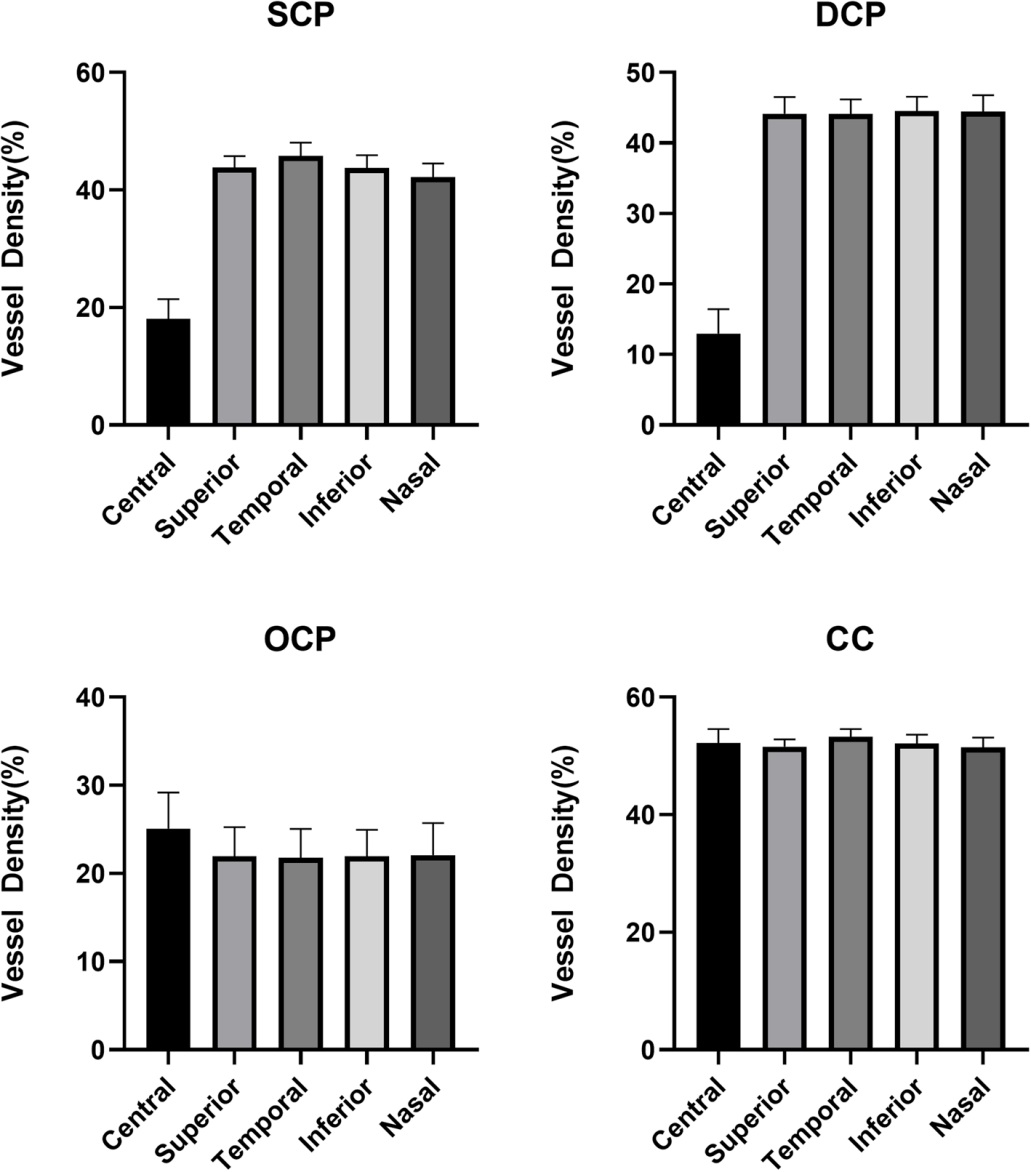
Macular VDs in the central and parafoveal subfields, respectively, measured in the different retinal plexuses. Abbreviations: SCP: superficial capillary plexus, DCP: deep capillary plexus, OCP: outer capillary plexus, CC: choriocapillaris

The VDs of different macular regions in each capillary plexus were reported respectively. At the level of SCP, there was no significant difference in VD between superior and inferior (*p* = 0.535), but there were significant differences in other regions when compared pairwise (all p < 0.001). At the level of DCP, the VD in the central region was significantly different from that in other regions (all p < 0.001), and the VD in the temporal region was significantly different from that in the inferior (*p* = 0.005) and nasal (*p* = 0.021) regions. At the level of OCP, there were significant differences between the VD of the central region and the other regions (all p < 0.001) and there was a significant difference in VD between the inferior and nasal (*p* = 0.001), and between the superior and nasal (*p* = 0.004). At the level of CC, there was no significant difference in VD between superior and nasal (*p* = 1.000), nor between central and inferior (*p* = 1.000). However, there were significant differences between the other regions when compared pairwise (all *p* < 0.001) (Fig. 3).

The results of correlation analysis are shown in Table 3. Age (*R* = -0.165, *p* < 0.001) was negatively correlated with VD of SCP. GCL average thickness (*R* = 0.077, *p* = 0.003) and choroid average thickness (*R* = 0.104, *p* < 0.001) were positively correlated with VD of SCP, but after age and sex adjustment, only choroid average thickness (*R* = 0.067, *p* = 0.010) was still positively correlated with VD of SCP. Sex (*R* = 0.123, *p* < 0.001) was correlated with VD of DCP, age (*R* = -0.201, *p* < 0.001) was negatively correlated with VD of DCP. Retina average thickness (*R* = 0.071, *p* = 0.006), GCL average thickness (*R* = 0.063, *p* = 0.016) and choroid average thickness (*R* = 0.144, *p* < 0.001) were positively correlated with VD of DCP. After adjusting for age and sex, there was only choroid average thickness (*R* = 0.108, *p* < 0.001) still positively correlated with VD of DCP. At the level of OCP, sex was correlated with the VD (*R* = 0.058, *p* = 0.027), age (*R* = 0.099, *p* < 0.001) was positively correlated with the VD, choroid average thickness (*R* = -0.056, *p* = 0.010) was negatively correlated with the VD. But, after adjusting for age and sex, choroid average thickness (*R* = -0.028, *p* = 0.293) was no longer correlated with the VD and GCL + average thickness (*R* = 0.072, *p* = 0.006) was positively correlated with the VD. At the level of CC, age (*R* = -0.088, *p* < 0.001) was negatively correlated with the VD, BCVA (*R* = 0.063, *p* = 0.017) and choroid average thickness (*R* = 0.068, *p* = 0.010) were positively correlated with the VD. However, after adjusting for age and sex, only BCVA (*R* = 0.082, *p* = 0.002) was positively correlated with the VD. After adjusting for age, it was found that sex was still correlated with the VD of DCP (*R* = 0.086, *p* < 0.001) and OCP (*R* = 0.080, *p* = 0.002). After adjusting for sex, age was still negatively correlated with the VDs of SCP, DCP and CC, and positively correlated with the VD (Table 4).

**Table 3:** Correlation analysis of ocular parameters and systemic parameters with macular VDs of different plexuses in the study sample

Parameters	SCP	DCP	OCP	CC
	R	R	R	R
Age (years)	-0.165***	-0.201***	0.099***	-0.088***
Sex	0.024	0.123***	0.058*	0.019
Diastolic pressure (mmHg)	-0.031	-0.071**	0.022	0.018
Systolic pressure (mmHg)	0.033	-0.044	-0.014	0.021
BMI (kg/m2)	0.023	-0.002	-0.038	0.036
BCVA (logMAR)	-0.051	0.022	0.010	0.063*
SE (D)	-0.026	-0.054	0.041	0.030
AL (mm)	-0.006	-0.024	-0.044	-0.049
IOP (mmHg)	0.111	0.041	0.006	0.075
RNFL thickness (μm)	0.027	-0.023	-0.006	-0.028
Retina average thickness (μm)	0.049	0.071*	0.005	0.016
GCL average thickness (μm)	0.077**	0.063*	0.046	0.008
Choroid average thickness (μm)	0.104***	0.144***	-0.056*	0.068*

^*^p ≤ 0.05 **p ≤ 0.01 ***p ≤ 0.001

*Abbreviations*: *BMI* Body mass index, *BCVA* Best-corrected visual acuity, *SE* Spherical equivalent, *AL* Axial length, *IOP* Intraocular pressure, *RNFL* Retinal nerve fiber layer, *GCL* Ganglion cell layer, *SCP* superficial capillary plexus, *DCP* Deep capillary plexus, *OCP* Outer capillary plexus, *CC* choriocapillaris

**Table 4:** Partial correlation analysis of ocular parameters and systemic parameters with macular VDs of different plexuses in the study sample after adjusting age and sex

Parameters	SCP	DCP	OCP	CC
	R	R	R	R
Age (years)	-0.163***	-0.181***	0.113***	-0.086***
Sex	0.001	0.086***	0.080**	-0.010
Diastolic pressure (mmHg)	0.002	-0.028	0.007	0.048
Systolic pressure (mmHg)	0.033	-0.017	0.001	0.051
BMI (kg/m2)	0.027	0.011	-0.040	0.032
BCVA (logMAR)	-0.018	0.052	-0.016	0.082**
SE (D)	-0.002	-0.024	0.034	0.036
AL (mm)	-0.046	-0.040	-0.050	-0.045
IOP (mmHg)	-0.104	-0.034	0.006	0.078
RNFL thickness (μm)	0.019	-0.032	-0.001	-0.032
Retina average thickness (μm)	0.018	0.044	0.033	-0.001
GCL average thickness (μm)	0.044	0.024	0.072**	0.011
Choroid average thickness (μm)	0.067**	0.108***	-0.028	0.048

^*^p ≤ 0.05 **p ≤ 0.01 ***p ≤ 0.001

*Abbreviations*: *BMI* Body mass index, *BCVA* Best-corrected visual acuity, *SE* Spherical equivalent, *AL* Axial length, *IOP* Intraocular pressure, *RNFL* Retinal nerve fiber layer, *GCL* Ganglion cell layer, *SCP* Superficial capillary plexus, *DCP* Deep capillary plexus, *OCP* outer capillary plexus, *CC* choriocapillaris

## Discussion

OCTA has recently become a useful imaging technique for providing robust, highly reproducible and high-resolution cross-sectional images and valuable anatomic information on the macular vessel. Before the large-scale application of OCTA technology in clinics, it is difficult to obtain standardized data of macular VDs. OCTA has been successfully proven to be able to qualitatively study the microvascular changes of diabetic retinopathy, retinal vein occlusion, arterial occlusion, glaucoma and other retinal and choroidal diseases [18–20]. So far, there are few references to macular VD values measured by OCTA in the literature, and the main determinants of VD values have not been determined [14, 16, 21, 22]. Our study filled the gap in the study of VD measured by OCTA in the Chinese elderly healthy population, established a normative database of VD in Chinese healthy elderly population, and analyzed the relationship between VD of different capillary plexus and other ocular parameters or systemic parameters.

Our data revealed VDs were 43.9% (3.2%), 44.3% (2.8%), 21.9% (5.9%), 52.1% (1.4%) in the SCP, DCP, OCP and CC. Because our study participants were the elderly population, the reported VDs are lower than those reported in the previous literature. Anna Dastiridou et al. reported that the mean VDs of SCP, DCP, CC in a population with an average age of 48.2 was 51.0% ± 3.1%, 54.0% ± 5.9%, 67.8% ± 2.6%, respectively [23]. Similarly, in a study with participants' average age of 48.3, Florence Coscas et al. reported that mean VDs of SCP and DCP were 52.58% ± 3.22% and 57.87% ± 2.82%, respectively [16]. A study in Turkey reported that mean VDs of SCP and DCP in participants older than 60 were 41.25% ± 2.51% and 36.01% ± 5.07% [12]. Yanan Dong et al. [24]reported that the mean VD values of SCP ( 44.3% ± 3.5% and 43.5% ± 3.8%) and DCP ( 44.7% ± 4.9% and 43.7% ± 4.8%) in participants under 80 years old were higher than those in participants over 80 years old.We speculate that this difference in the senior participants may be due to the measurements made with different OCTA devices.

Our study also found a significant negative relation between age and VD in the SCP, DCP and CC that is in line with some of the findings reported in previous studies [1, 15, 23, 25, 26]. The discovery of OCTA is consistent with the anatomical trend of human retinal structure with age that is the retinal capillary density of the elderly decreases significantly [27]. With the increase of age, the metabolic activity of human brain tissue begins to weaken. As a continuation of the central nervous system, the metabolic activity of the eye also begins to weaken [28]. The decrease of VD in different capillary plexus of macular area may reflect this metabolic change of aging. According to Coscas et al., after the age of 60 years, VD is higher in women than men [16], similar results were found in our study. However, this difference was not shown in some studies with younger participants [13, 14], probably due to late vascular aging in females.

Our study found that there were differences in VD in different regions of the same macular capillary plexus. However, the differences we found were different from the results reported by Florence Coscas et al. Their study found that the superior and inferior regions mean VDs were higher (*P* < 0.001) than those in the temporal and nasal regions in both SCP and DCP plexuses [16], but there was no such obvious trend in our study results. These results are also inconsistent with the result of Hayreh [29] on the watershed zones: Blood flow to the temporal retina was approximately three times larger than that to the nasal retina, with no difference between the superior and inferior retina. We think that the differences between different regions could be related to the difference in metabolism in different regions of the macula.

In this study, we found that the choroid average thickness was positively correlated with VDs of SCP and DCP. This result is consistent with the conclusions of Al-Sheikh et al. [30] The capillary network of the retina is the basis of maintaining retinal nutrition. The density or morphological change of the capillary network of the retina will affect the homeostasis of the retina [31]. We speculate that the relationship between VD and choroid average thickness may be related to the nutritional function of capillaries. We also found that VD in CC was positively correlated with BCVA (logMAR), which means the greater the VD in CC was, the worse the visual acuity was. The choroid is responsible for supplying nutrients to the outer retina, and the layer of rods and cones is located in the outer retina [32]. Therefore, the relationship between BCVA and VD in CC may be caused by the compensatory increase of choroidal capillaries caused by insufficient blood supply in the layer of rods and cones.

Our study had several strengths. Firstly, it was one of the largest studies to date of OCTA retinal measures among Chinese urban healthy elderly participants. Secondly, we used OCTA 6 × 6-mm line scan mode to obtain a wider view field of macula, and all included OCTA images had a high quality (signal strength index ≥ 40). Thirdly, we divided macular area into different regions according to ETDRS and then we analyzed the differences in different regions of different capillary layers. Finally, the measurement of all parameters in this study follows a strict, prespecified principle, and all processes are operated by professionals to ensure the reliability of the data. However, our findings are subject to some limitations. Firstly, this study is a cross-sectional study and cannot assess the causal relationship between those parameters. Secondly, this study only included elderly participants and could not obtain the standard value of VD measured by OCTA in a longer age range which limits the transferability of the present results. Thirdly, there is a significant difference in sex proportion among the included research subjects, with men accounting for only 30.4% of the study population. Finally, the segmentation of OCTA images in this experiment depends on the built-in software of Triton, but at present, the correct segmentation of images by this software is only possible for normal macula. This method may lead to wrong image segmentation [4].

In conclusion, this study analyzed VD measured by OCTA in a large Chinese urban healthy elderly population, providing an example for the establishment of a normative database of VD in the future. In this study, we also explored the related factors of different capillary plexus, and provided new ideas for the future study of the relationship between macular VD and disease.

## Data Availability

The data that support the findings of this study are available from the corresponding author, Juping Liu, upon reasonable request.

## Abbreviations

VD: Vessel density
OCTA: Optical coherence tomography angiography
FFA: Fundus fluorescein angiography
DR: Diabetic retinopathy
AMD: Age-related macular degeneration
SCP: Superficial capillary plexus
DCP: Deep capillary plexus
OCP: Outer capillary plexus
CC: Choriocapillaris
BCVA: Best-corrected visual acuity
logMAR: Logarithmic minimum angle of resolution
SE: Spherical equivalent
IOP: Intraocular pressure
AL: Axial length
SLO: Nonmydriatic 200° ultra-wide-field scanning laser ophthalmoscopy
ILM: Internal limiting membrane
IPL: Inner plexiform layer
INL: Inner nuclear layer
RPE: Retinal pigment epithelium
BM: Basement membrane
ETDRS: Early treatment diabetic retinopathy study
SS-OCT: Swept-source optical coherence tomography
RNFL: Retinal nerve fiber layer
GCL: Ganglion cell layer
BMI: Body mass index
